# Retraction: The complete mitochondrial genomes of the nematode-trapping fungus *Dactylellina cionopaga*.

**DOI:** 10.1080/23802359.2019.1701829

**Published:** 2019-12-18

We, the Editor and Publisher of Mitochondrial DNA Part B: Resources, have retracted the following article:

Chu Deng & Ze-Fen Yu (2019) The complete mitochondrial genomes of the nematode-trapping fungus Dactylellina cionopaga *Mitochondrial DNA Part B: Resources*, 3:1, 419–423. https://doi.org/10.1080/23802359.2019.1573115

This article has been retracted as the accession number reported in this article, through the following correction notice, has been found to be unverified.

(2019) Correction, Mitochondrial DNA Part B: Resources, 4:1, 1486 https://doi.org/10.1080/23802359.2019.1603877

We have not been able to resolve these concerns with the authors, who have been unable to provide a verified Genbank accession number, and therefore retract this article.

We have been informed in our decision-making by our policy on publishing ethics and integrity and the COPE guidelines on retractions.

The retracted article will remain online to maintain the scholarly record, but it will be digitally watermarked on each page as “Retracted”.

